# Recent Developments and Formulations for Hydrophobic Modification of Carrageenan Bionanocomposites

**DOI:** 10.3390/polym15071650

**Published:** 2023-03-26

**Authors:** Rubie Mavelil-Sam, Elizabeth Mariya Ouseph, Marco Morreale, Roberto Scaffaro, Sabu Thomas

**Affiliations:** 1 School of Nanoscience and Nanotechnology, Mahatma Gandhi University, Kottayam 686 560, India; rubiemsams@gmail.com; 2 School of Energy Materials, Mahatma Gandhi University, Kottayam 686 560, India; elizabethmariyaouseph@gmail.com; 3 Faculty of Engineering and Architecture, Kore University of Enna, 94100 Enna, Italy; 4 Department of Engineering, University of Palermo, Viale delle Scienze, 90128 Palermo, Italy; 5 Department of Chemical Sciences, University of Johannesburg, P.O. Box 17011, Doornfontein, Johannesburg 2028, South Africa; 6 Institute of Biophysics of the Siberian Branch of the Russian Academy of Sciences, Siberian Federal University, 79 Svobodnyi Av., Krasnoyarsk 660041, Russia; 7 International and Inter-University Centre for Nanoscience and Nanotechnology (IIUCNN), Mahatma Gandhi University, Kottayam 686 650, India

**Keywords:** carrageenan, bionanocomposites, hydrophobicity, nanofillers, bioactive agents

## Abstract

Versatility of the anionic algal polysaccharide carrageenan has long been discussed and explored, especially for their affinity towards water molecules. While this feature is advantageous in certain applications such as water remediation, wound healing, etc., the usefulness of this biopolymer is extremely limited when it comes to applications such as food packaging. Scientists around the globe are carrying out research works on venturing diverse methods to integrate hydrophobic nature into these polysaccharides without compromising their other functionalities. Considering these foregoing studies, this review is designed to have an in-depth understanding of diverse methods and techniques adopted for tuning the hydrophobic nature of carrageenan-based bionanocomposites, both via surface alterations or by changes made to their chemical structure and attached functional groups. This review article mainly focuses on how the hydrophobicity of carrageenan bionanocomposites varies as a function of the type and refinement of carrageenan, and with the incorporation of additives including plasticisers, nanofillers, bioactive agents, etc. Incorporation of nanofillers such as polysaccharide-based nanoparticles, nanoclays, bioceramic and mineral based nanoparticles, carbon dots and nanotubes, metal oxide nanoparticles, etc., along with their synergistic effects in hybrid bionanocomposites are also dealt with in this comprehensive review article.

## 1. Introduction

In a type of hybrid material known as bio-nanocomposites, at least one of the dimensions must be less than 100 nm and is reinforced by nanoscale components. Over the years, cheaper prices for conventional plastics have forced biodegradable and bio-based plastics to compete, and bionanocomposites stand out as one of the most promising options due to their availability, lower density, cost effectiveness, and exceptional properties such as positive environmental advantages with regard to their final disposal and raw material consumption. Environmentally friendly plastics should be urgently employed to address this issue as ecological concerns and the high demand for advanced polymeric materials with balanced properties have resulted in the development of hybrid composites (incorporating two or more diverse reinforcement materials/fibres) [1,2,3,4].

Natural biopolymers have increasingly found application as matrices in bionanocomposites over the past few years. Bio-based polymers are polymeric materials derived from renewable resources such as agricultural waste, food processing industry waste, and other types of natural materials (animals/plants/algae). The usage of such polysaccharides allows for the homogeneous dissemination of natural filler particles, particularly nanoparticles inside the matrix as well as effective stability parameters. Among such biopolymers, seaweed-based biomaterials and polysaccharides have garnered a lot of interest in recent years [5,6].

In the present world where more concern is given to environmental protection, biopolymers have found immense importance due to their eco-friendly nature. Carrageenan is one such explored material that has been found to have great potential for applications in a variety of fields such as the food industry (both as a food ingredient and as plastic alternatives in food packaging) and biomedical fields (wound healing, drug delivery, tissue engineering and regenerative medicine) [7,8]. Carrageenan is one of the most promising and appealing polysaccharides generated from seaweeds, which is a renewable bioresource that possesses exceptional composite mechanical properties [9,10]. They are among the oldest and most popular natural polysaccharides as they are biocompatible, biodegradable, affordable, and safe. Carrageenan has been traditionally used as food additives in the form of thickening, stabilising, and emulsifying agents, while its other applications include cosmetics, agriculture, superabsorbent materials, energy storage, water remediation, and electrochemical applications [11,12,13,14,15]. Since the packaging industry is one of the major contributors to pollution due to the extensive use of plastics, researchers have successfully developed green alternatives to plastics, among which carrageenan has been found to be a suitable replacement material [16,17]. As this polysaccharide also has certain innate biomedical properties such as antiviral, antitumor, anticoagulant, anti-inflammatory, etc., it has also been proven to be a potential candidate for applications such as wound healing, drug delivery, and so on [18,19]. Interestingly, carrageenan is also applied in tissue engineering and regenerative medicine due to its viscoelastic property [20,21]. It depicts high gel-forming properties, even in the absence of ions, which makes it a superior material when compared to its counterparts such as alginate. Additionally, carrageenan is an inexpensive polymer, thanks to its simple extraction processes. It is also non-toxic and rather biocompatible, posing no harm for handling. Furthermore, due to its ability to dissolve in water, carrageenan is used to thicken (improve the viscosity of) aqueous solutions, build bionanocomposites in the form of hydrogels with variable degrees of hardness, prepare composite films, or even create aerogels depending on their intended uses [5].

Despite the various advantages of carrageenan, which are attributed to its extensive application as a food additive, several safety concerns have been raised. Researchers claim the potential possibility of poligeenan (i.e., degraded carrageenan), generated by the hydrolysis of carrageenan, to be a carcinogen, which has also been found to cause ulcerations, colitis, and neoplasm [22]. Around the same time, in a review article by Tobacman [23], the author strongly suggested that carrageenan intake (both degraded and undegraded) could cause ulcerative colitis (intestinal inflammations) and intestinal neoplasms, and that degraded carrageenan could be a potential carcinogen, causing cancers in animal models.

A few studies have also suggested that carrageenan can cause intestinal bowel disease (IBD), diabetes, or even cancer. In 2018, David et al. [24] reported that long-term exposure to carrageenan could have toxic effects on human health, and that carrageenan could cause various health concerns such as an increase in colonic tumours, promotion of aberrant crypt foci, increase in small intestine tumours, higher cell proliferation in the colon crypts, ulcerations of the cecum and large intestine, and epithelial cell loss. Additionally, excessive exposure to this polymer could be a possible cause leading to glucose intolerance. The study by Bhattacharya et al. in 2017 [25] confirmed the potential of carrageenan to cause ulcerative colitis, and a review by Martino and co-workers around the similar time period reported the inflammatory effects of carrageenan exposure, confirming the impact of carrageenan in the disruption of the epithelial barrier, and dysregulation of the immune response to the gut microbiome has repeatedly been implicated as well as being a cause of IBD [26]. A review by Liu et al. [27] provides an extensive information on the various health effects of prolonged exposure to carrageenan, where they reported various instances where evidence generated in various animal models and clinical trials in humans suggested that the long-term intake of food-grade carrageenan, particularly those with the random coil conformation, may increase the incidence of intestinal inflammation or promote inflammatory recurrence in patients with colitis. The effects of carrageenan were analysed by Pogozhykh and team [28] via routine histological studies and also by examining the inflammatory marker levels, where they found that the oral administration of E407a (semi-refined carrageenan) was found to be associated with altered small and large intestinal morphology, infiltration of the lamina propria in the small intestine with macrophages, high systemic levels of inflammation markers, and changes in the lipid order of the phospholipid bilayer in the cell membranes of leukocytes, alongside the activation of their apoptosis, hence suggesting that oral exposure to E407a through rats results in the development of intestinal inflammation.

Though many of these findings have not been confirmed by means of human trials, significant concerns have been made in this regard and numerous debates continue to take place regarding the safety of carrageenan as a food additive. The EFSA (European Food Safety Authority) panel published a report in 2018 [29] where they observed no chronic toxicity, carcinogenicity, or genotoxicity pertaining to carrageenan use while also considering the existence of certain uncertainties regarding the chemistry, exposure assessment data, and toxicological and biological data. This led the panel to suggest that the present acceptable daily intake of carrageenan and processed Eucheuma seaweed (75 mg/kg by weight per day) should not be completely relied on, but rather considered as temporary.

As a continuation to the above report, EFSA also called for the collection of technical and toxicological data on carrageenan (E 407) for uses in foods for all population groups including infants below 16 weeks of age, since the risk assessment approach followed at the time by the former EFSA Panel on Food Additives and Nutrient Sources added to Food (ANS Panel) in the re-evaluation of food additives did not apply to this age group, as well as to address the data gaps that have been identified for all population groups in the already published EFSA opinion on this food additive [30]. The basic rationale of this data collection was to address the uncertainties regarding the incorporation of carrageenan in food. The main aspects to be addressed through this included both toxicological and technical data; the role of sulphate and its interactions with gut microbiota, chronic toxicity and developmental toxicity, inflammatory bowel diseases, induction of glucose intolerance, fate and reactions products of carrageenan, acceptable levels of usage, etc., which have been debated throughout the years.

Additionally, elaborate studies have been carried out by several scientists in recent years, among which a study conducted by Alli and team regarding the in vitro toxicity of carrageenan on oral cavity cells and tissues stated that no significant detrimental effects were observed in the cell number and viability [31]. Valado and group studied the effect of carrageenan on cardiovascular disease (CVD) and metabolic syndrome, where they reported that interestingly, carrageenan has the potential to decrease the lipid profile in the system, thereby preventing CVD and controlling metabolic syndrome [32]. Additionally, Borsani and co-workers reported that not much data were available to claim the safety concerns of carrageenan with inflammatory bowel diseases and allergic reactions [33]. Similar conclusions were also drawn from the analysis carried out to study the safety and efficiency of carrageenan as a feed additive for pets [34]. The International Agency for Research on Cancer (IARC) has classified degraded carrageenan as 2B, a possible human carcinogen, based on animal study data, whereas they classified native carrageenan as 3, indicating a non-classification pertaining to carcinogenicity in humans [22]. Since carrageenan is generally used in foods at significantly low doses such as 0.1 to 2%, the Joint FAO/WHO Expert Committee on Food Additives (JECFA) suggested that carrageenan intake corresponding to its use as food additives was by no means a safety concern. They also concluded that the use of carrageenan in infant formula foods or for special medical purposes at concentrations as high as 1000 mg/L was of no alarm. Additionally, the U.S. Food and Drug Administration suggested that the carrageenan levels used in foods to attain various functionalities were harmless and that no upper limit of usage needed to be considered [35]. Even though carrageenan has been traditionally used as a food additive for various applications and not much data from the literature is available regarding the safety issues associated with carrageenan, it is advisable that its consumption be retained within a limited quantity.

**Figure 1:**
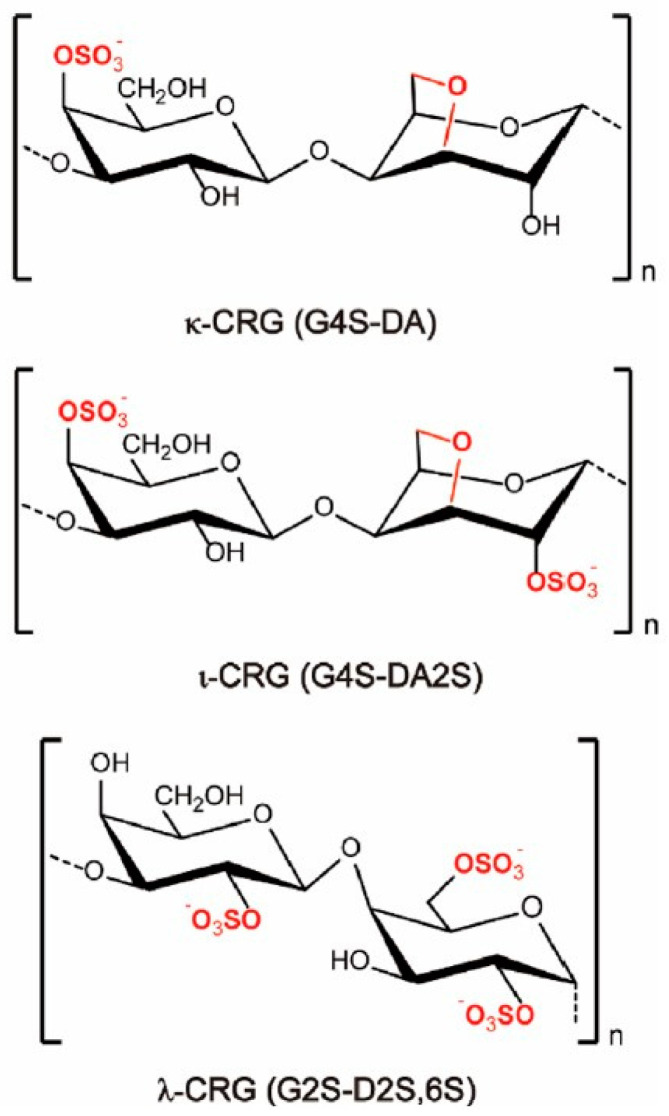
Idealised chemical structures of κ-, ι-, and λ-CRG. G, DA, and D refer to the β-D-Galp, 3,6-anhydro-α-D-Galp, and α-D-Galp units, respectively (taken from [38] with permission from Elsevier).

Generally, carrageenans are composed of linear chains of galactans, and depending on the amount of sulphate present, they can be divided into three types: kappa (κ, 20%), iota (ɩ, 33%), and lambda (λ, 41%) (Figure 1). They are distinctive in that they have both ionic and hydrophilic characteristics, the former of which influences the latter, and opens up intriguing possibilities for a variety of uses. However, because of their low vapour barrier qualities and hygroscopic and hydrophilic nature, their application is relatively restricted. It is therefore necessary to add a hydrophobic component to boost its water barrier property, especially in humid environments [4,36,37].

Kappa carrageenan, being a water-soluble sulphated polysaccharide produced from the red algae *Kappaphycus alvarezii*, consists of a long linear chain of D-anhydrogalactose and D-galactose attached to an anionic sulphate group (−OSO_3_−). A quicker and more affordable red algae extraction process could provide semi-refined carrageenan (SRC), another renewable resource that is cost effective and commercially available. A paucity of research has been performed on semi-refined carrageenan-based films due to various impurities that alter the mechanical and optical characteristics of the film. When specific ions such as calcium (Ca^2+^) and potassium (K^+^) are present, carrageenan has exhibited easy ionic cross-linking [2,3,5,39,40,41,42].

**Figure 2:**
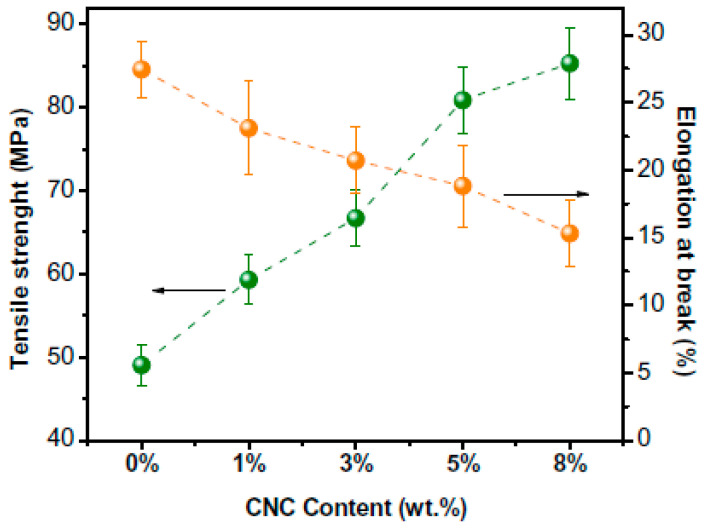
Tensile strength and elongation at break of the kappa–carrageenan (KC) composites with cellulose nanocrystals (CNC, 1 to 8 wt.%) (taken from [9] with permission from Elsevier).

Numerous research works have been conducted to adapt the functions of kappa carrageenan to overcome its inferior properties such as low tensile strength and poor barrier characteristics, which limit their practical usage. It has been demonstrated that incorporating nanofillers or plasticising reagents is crucial to enhancing the sorption capabilities of hydrophilic polymeric materials [43]. One method is to use nanoclays, nanofibres, and nanocrystals as nanofillers to reinforce the biodegradable polymers [39]. The mechanical, water vapour barrier, and UV barrier qualities of carrageenan films can all be enhanced by the addition of beneficial nanomaterials [40]. One such proof was provided by the study team led by Kassab, who looked at how adding cellulose nanocrystals affected the mechanical properties of composites made of kappa–carrageenan (Figure 2) [9].

Due to their unique size-dependent optical, mechanical, chemical, electrical, and magnetic properties, which fundamentally differ from those of bulk materials, inorganic nanoparticles have already established themselves as an essential component for many industrial applications [5]. Due to cellulose’s renewability, relatively high strengths and moduli, excellent adhesion, remarkably high aspect ratio, lighter weight and biodegradability, bio-based or organic fillers such as cellulose nanocrystals (CNCs) have attracted significant interest within the scientific community. Organoclays are among the less expensive inorganic nanoparticles since they are produced from easily accessible environmental resources and are commonly available in the market [4]. The shape, structure, and physical characteristics of the resulting composites such as tensile strength, elongation, and hydrophobicity can all be altered by incorporating metal oxide nanoparticles, nanotubes, and carbon dots to suitable biopolymers [5].

For the further development of polymeric materials with the desired features, the sorption properties of biomaterials may be a significant component. Water molecules enter the polymeric matrix through physical adsorption, chemisorption, and condensation during moisture absorption. In order to assess the surface wettability and surface hydrophobicity characteristics of polymer films, the water contact angle (WCA) is typically measured. The WCA value of neat films of carrageenan typically ranges between 60 and 65 degrees, indicating that it has a hydrophilic surface, as in general, biopolymer films with a WCA value of less than 65 degrees are regarded as hydrophilic [42,44,45]. It is a major factor that determines how well nanocomposite films work, particularly when they are used to package fresh food produce and other edible products, where they must come in touch with wet surfaces and conditions. Purity, the proportion of amorphous to crystalline regions, hydrophilic/hydrophobic ratio, and the mobility of the polymeric chain are all factors that affect the water vapour sorption and permeability of bionanocomposites [36,43]. Film solubility can be useful in circumstances where the films will be consumed alongside the hot product. It can also play a significant role in determining how quickly a film degrades when used as a packaging wrap system. A decrease in water sorption, on the other hand, makes it possible to produce products of higher quality because the quality of packing materials is thought to be inversely proportional to their water vapour permeability [5,37]. Additionally, a reduced water solubility of hydrophilic materials is particularly crucial since most food applications call for a specific level of water resistance to prevent film disintegration when they come into contact with moist food surfaces such as meat, dairy, and freshly cut fruits and vegetables. Outstanding tensile and elongation properties have also been reported to be necessary for carrying and distributing packages in good condition, while superior barrier properties serve to avoid oxidative alterations in the food products, thereby keeping its freshness [36,39,43].

In light of the aforementioned backdrop, this review is designed to address the factors affecting the hydrophobicity of carrageenan-based bionanocomposites, with special emphasis on the contribution of the matrix (types of carrageenan), effect of plasticisers as well as the role of various types of nanofillers including nanoparticles based on polysaccharides, minerals, metal oxides, carbon dots, and bioactive agents. Hence, this review paper as a whole has been brought together with the hope of using it for better understanding and further advancement in the field of carrageenan-based bionanocomposites.

## 2. Research Strategy and Data Collection

**Figure 3:**
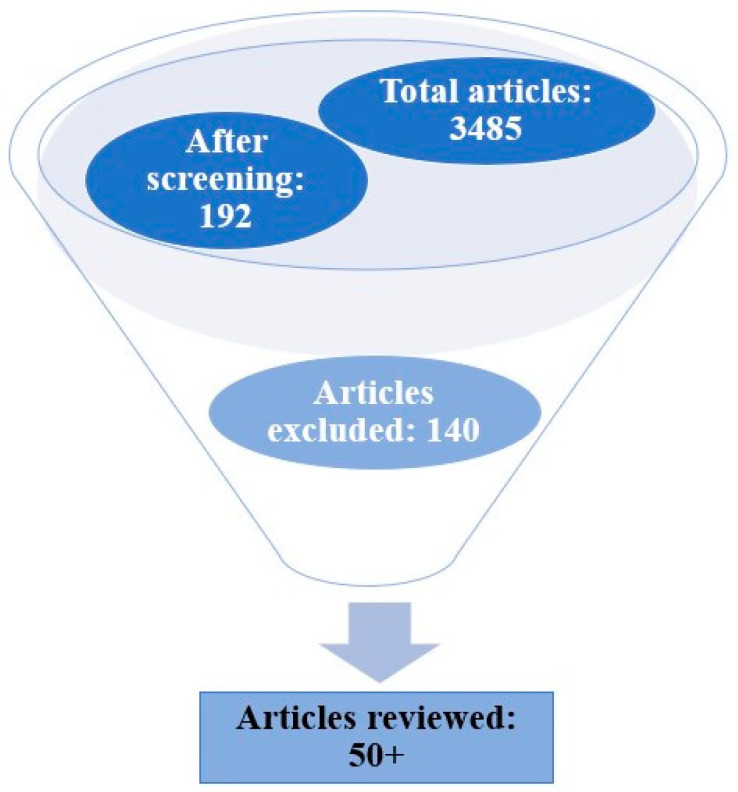
Search strategy flowchart adopted for the review.

This comprehensive review on the theme hydrophobic modification techniques of carrageenan-based bionanocomposites is an area of enormous research with great relevance in the present scenario. In this article, we discuss in detail the various protocols and formulations for augmenting the hydrophobic nature of bionanocomposites with carrageenan as the matrix, for which we reviewed several articles persistent to this topic. The relevant resources were extracted from the literature with the help of search engines such as Google Scholar, Science Direct, Bielefeld Academic Search Engine (BASE), PubMed.gov, and Semantic Scholar. The main search strategy adopted was database searches for carrageenan, hydrophobic modification, bionanocomposites, nanofillers, bioactive agents, etc. as the keywords (Table 1). The criteria for selecting articles mainly included the time of publication (2018–2023), impact factor (higher than 4.9), access to full text, etc. All of the collected data were screened to discard irrelevant articles for this study (Figure 3). The extensive data search revealed several publications that ranged up to 192 articles after screening, out of which only relevant articles/work papers were reviewed, which summed up to above 50. About 140 articles were avoided as they fell under the category of review articles, citations, repetitions, etc.

In this review article, since we focused on carrageenan-based bionanocomposites, other formulations such as nanocomposites, biocomposites, and blends were filtered out. We also came across a handful of duplicates that were excluded. The main articles referenced in this study were from high-impact journals such as *Food Hydrocolloids* (11.504), *Carbohydrate Polymers* (10.723), *Food Packaging and Shelf Life* (8.749), *International Journal of Biological Macromolecules* (8.025), MDPI *Marine Drugs* (6.085), MDPI *Nanomaterials* (5.719), MDPI *Polymers* (4.967), *Polymer Testing* (4.931), etc. During the research and data collection, we adopted Mendeley reference manager to store, manage, and cite the gathered references, and the research strategy adopted is believed to be suitable and effective as it helped to obtain all possible data to date in related fields.

## 3. Factors Affecting Hydrophobicity of Carrageenan Bionanocomposites

### 3.1. Role of Matrix/Carrageenan Type

#### 3.1.1. Kappa vs. Iota

As mentioned earlier in the introduction, two major carrageenan types comprise of kappa and iota variants. While the former consists of one sulphate group in the glycosidic chains, the latter consists of two sulphate groups. It has also been widely reported that kappa-carrageenan gives firmer gels and composites in comparison to its iota counterpart [46,47,48,49]. In the case of kappa-carrageenan, incorporation of zinc oxide nanoparticles (ZnO NPs) significantly increased the solubility of the resultant bionanocomposite film. However, a further increase in the ZnO concentration resulted in a decrease in the solubility of bionanocomposites, which can be attributed to the linkages between ZnO and kappa-carrageenan (Table 2). An increase in the quantity of nanoparticles (ZnO) in the films might have caused more hydrogen bonds to form between the components of kappa-carrageenan and ZnO, and in comparison to the control films, unbound water molecules would not have interacted with the nanocomposite films as strongly. The authors hypothesised that the solubility of bionanocomposite films may have been impacted by the stability of the nanoparticles, especially at various concentrations. In addition to the size and surface area, other elements such as the surface curvature and nanoparticle roughness are also thought to affect how the solutions behaved. Since the concentration of ZnO NPs had a significant impact on the dissolution parameters of bionanocomposite films, the solubility index of the films with lower ZnO NP concentrations was higher [5]. Iota-carrageenan films containing silicon dioxide and zinc oxide nanoparticles (SiO_2_–ZnO NPs) showed the lowest water vapour permeability (WVP), which was correlated with the SiO_2_–ZnO nanoparticle performance, according to Praseptiangga and colleagues. Due to the water-soluble properties of iota-carrageenan associated with the sulphate ester group, the water solubility of these films was considerably (*p* < 0.05) higher than that of the other films with varied compositions [2].

#### 3.1.2. Refined vs. Semi-Refined

As a function of the degree, extent, and stage of extraction and processing methods, carrageenans are classified as refined and semi-refined carrageenans (RC and SRC, respectively), the key variance among them being the presence of cellulosic residual materials in the latter type. Since there are fewer processing steps in the production of SRC, the global price is typically only two-thirds the price of conventional RC. For example, refined carrageenan costs around INR 1005/kg (USD 12.3/kg), whereas the semi-refined type would cost only around INR 660/kg (USD 8/kg) [50]. While the refined type is widely used in the food, pharmaceutical, and cosmetic industries, SRC is primarily utilised for other applications such as food packaging, which do not require such a high level of refinement [51].

A few research groups have studied the effect of the extent of carrageenan refinement on the mechanical and physicochemical properties of the resultant composites, among which nanocellulose, nanoclays, and metal oxide nanoparticles have commonly been reported; a parallel comparison of which are shown in Table 3, Table 4 and Table 5, respectively.

In the case of all of the aforementioned fillers (nanocellulose, nanoclays, and ZnO NPs), a decrease in the water interactive properties of the composites could be observed, which is also fairly proportional to the filler concentration. At the same time, for carrageenan films incorporated with nanocellulose fibrils, the ones with semi-refined carrageenan showed a higher moisture uptake and water vapour permeability than their refined counterparts, which the authors attributed to the distribution of solid impurities (i.e., cellulose and minerals) within the structural matrix. However, on the other hand, higher contact angle values were exhibited by semi-refined composites that showed their hydrophobic nature [51].

**Figure 4:**
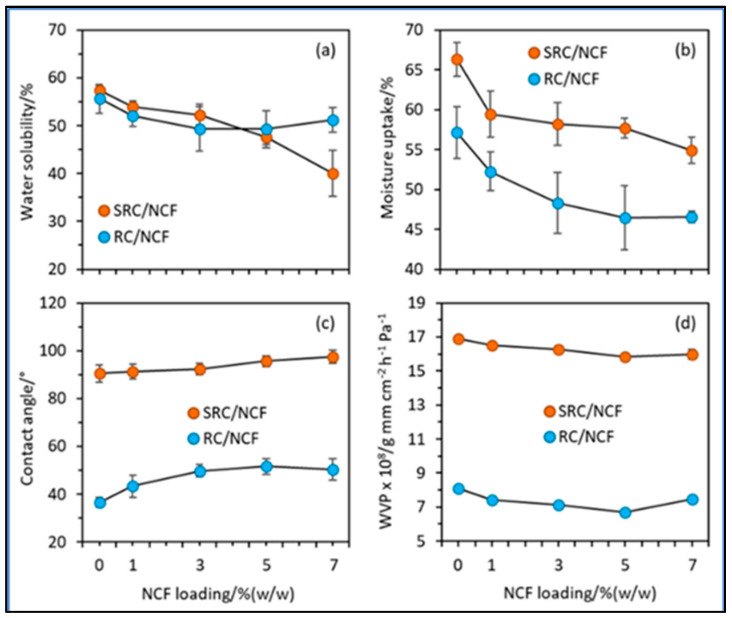
Plots of (**a**) water solubility, (**b**) moisture uptake, (**c**) contact angle, and (**d**) water vapour permeability (WVP) against the NCF loading for the SRC/NCF and RC/NCF film formulations (taken from [51], MDPI CC BY).

Due to the weaker compact matrix framework in the SRC formulation, it was also anticipated that impurities in the SRC material would cause inferior water resistance and barrier qualities in SRC/NCF films compared to RC/NCF films. However, whereas the integration of NCF above 5% had little impact on the characteristics of RC-based composites, a loading of up to 7% was observed to have an impact on the water interaction capabilities of SRCs (Figure 4). This could be the result of the interaction between the particulate impurities in SRC and NCF, which delayed the self-agglomeration of the NCF particulates within the SRC film framework [51].

### 3.2. Role of Plasticisers

The effect of plasticisers on carrageenan-based bionanocomposites has been investigated by various researchers over the years [37,55,58,59,60] and have shown interesting results on how the nature and procedure of the addition of plasticisers influenced the end result of the fabricated composites. One of the studies by Nouri and co-workers in 2018 reported that while carrageenan films without any additives showed a moisture content of 13.6%, it was increased to 31.8% after the addition of glycerol. This was attributed to the fact that the high hydrophilic nature of glycerol increases the amount of moisture in the resultant films [37]. Additionally, while conducting a comparative study on the effect of plasticiser types on various properties of carrageenan based composites, researchers have reported that when compared to sorbitol containing composites, films plasticised with glycerol exhibited a relatively higher moisture content and water solubility, but similar water vapour permeability values [61].

Another study was carried out to understand how the order of the addition of plasticiser affected the composite properties. Glycerol was added to the mixture via two different procedures P1 and P2 (P1: addition of glycerol after nanoclay; P2: addition of glycerol before nanoclay to carrageenan solution). It was observed that there were significant changes depending on the step of plasticiser addition, which directly affected the properties of the final nanocomposite films (Table 6). The water vapour permeability values were higher for composites prepared via P2 in the case of neat films and those containing 1% *Zataria multiflora* plant extract (ZME), whereas composites prepared via P1 exhibited higher WVP values when 2% and 3% ZME were added [55].

### 3.3. Effect of Nanofillers

#### 3.3.1. Polysaccharide-Based Nanofillers

Upon mixing kappa-carrageenan with cellulose nanocrystals (CNCs), the WVP of the composite films was found to decrease exponentially (from 3.21 to 2.25 g·mm/m^2^·day·kPa) with an increase in the CNC content (1 to 7%). This enhancement was attributed to the uniform dispersion of the nanofillers throughout the matrix, where the dispersed CNC induced a difficult path for water vapour, thereby hindering the diffusion of water molecules through the matrix [39].

In the instances of both the semi-refined and refined carrageenan (SRC and RC) composite films, the moisture uptake and water solubility were reduced and the contact angle improved with the inclusion and higher loading of nanocellulose fibrils (NCF). The association of the hydroxyl groups of NCF with the hydroxyl and/or carboxyl groups in the carrageenan backbone through substantial hydrogen bonds, which led to an enhancement in polymeric cohesiveness within the network, is another explanation for the improved characteristics of the SRC/NCF and RC/NCF films [51].

**Figure 5:**
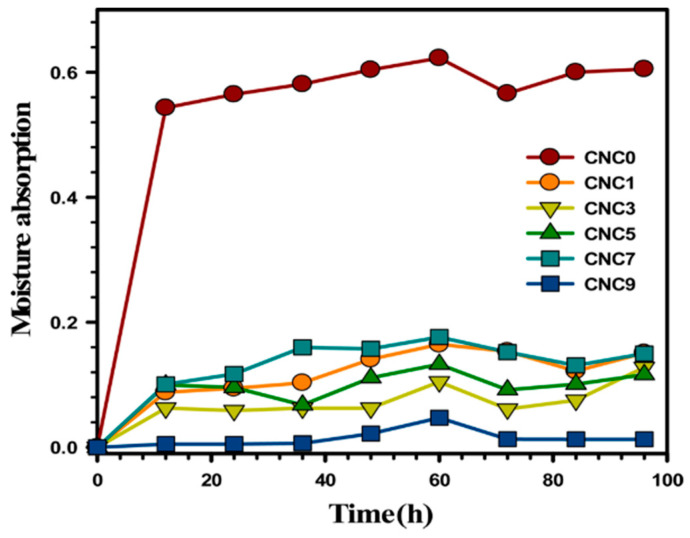
Trends in the moisture absorption of the kappa-carrageenan/nanocellulose composites at different loading (taken from [36] with permission from Elsevier).

In one of the research reports, Yadav and Chiu [36] revealed that the optimum rate of water absorption of the neat KC films dropped from 885.94% to 561.79% with the addition of CNCs. The lower water absorption was attributed to the higher crystalline content of cellulose than that of KC, which, when reinforced, functioned as a deterrent to the swelling of KC molecules, thereby causing the observed lowering in water absorption. Additionally, the 3D cellulose network of CNCs, which decreased the quantity of hydroxyl groups partaking in hydrogen bonding, was attributed to the cumulative drop in the hydrophilic nature of the nanocomposite film. Table 7 displays the impact of the CNC contents on the water interaction parameters of KC films, and Figure 5 illustrates the impact on moisture absorption.

The increased moisture absorption observed when the duration was extended from 12 h to 60 h was ascribed to the greater availability of −OH groups of KC. Beyond 60 h, the absorption of moisture was seen to decrease, which was due to the 3D cellulosic network, which restricted the chain mobility and reduced the number of hydroxyl groups. Hence, it can also be established that the equilibrium moisture uptake value depends on the hydrophilicity and morphology (macro-voids, free volume, crystal size, and crystallinity degree) of the resultant composites [36]. A decrease in the rate of water solubility (60.94% to 47.97%) was also observed with an increase in the CNC composition (0% to 9%). However, at 9 wt.% loading of CNC, the permeability was seen to increase due to higher chances of agglomeration at a high filler loading, which was also reported by other researchers [62,63].

#### 3.3.2. Nanoclay/Bioceramic/Mineral Nanoparticles

Thanks to their natural origin and affordable price, nanoclays are widely used as nanofillers for various polymers. The high modulus of clay platelets causes them to reduce the water-vapour permeability of polymer–clay composites, a crucial characteristic for biopolymer films and coatings [43,64,65]. The therapeutic efficacy of polymeric nanocomposite scaffolds has been shown to be improved by the addition of bioceramics, particularly hydroxyapatite (HA) (Ca_10_(PO_4_)_6_(OH)_2_). In order to build artificial bone, bioceramics and biopolymers are combined in a significant way. Over the past 20 years, hydroxyapatite (HA) has undergone substantial research as a material for bone replacement and repair. The bioceramic component of bioactive biopolymers serves as both crosslinking agents and bioactive factors, offering a flexible method to create multifaceted biologically active materials for applications in tissue engineering and wound healing [66,67,68].

Several research groups have investigated the role of diverse types of nanoclays with varying hydrophilic properties on the final water interactive characteristics of the resultant carrageenan composites (Table 8). Sedayu et al. (2021) reported a higher water vapour barrier of semi-refined carrageenan (SRC) films with an increase in nanoclay reinforcement. Their findings demonstrated how the barrier properties of the films were affected by the hydrophilicity of the clays with the presence of less hydrophilic clays, leading to a higher decline in WVP of the composite materials. In theory, the meandering path generated by the distribution of clay particles within the polymer matrix, which obstructs water vapour diffusion traversing through the film, is responsible for the lower WVP of composite films as a result of nanoclay incorporation [69]. In comparison to hydrophilic clays, the more hydrophobic clays clearly facilitated a more impenetrable layer, resulting in a highly efficient water vapour barrier. This was attributed to the fact that the hydrophobic nanoclay formulation in the polymer matrix had less hydrogen bonding or physical/chemical interaction with nearby water molecules, resulting in a lower WVP than those comprising the hydrophilic nanoclays [54,70].

In a work carried out by Dogaru and team [43], they synthesised nanocomposites composed of kappa-carrageenan and bentonite nanoclay (BT) with varying concentrations of each component (from 0 to 15%) and investigated the water sorption characteristics, in addition to other morphological and structural attributes. They demonstrated that all films gradually increased in moisture content (MC) with rising relative humidity (RH) in the surrounding environment. All films absorbed tiny amounts of water at reduced relative humidity (RH) levels. Higher BT content films resulted in more contact between the hydroxyl groups of the carrageenan chains and BT, which reduced the accessibility of hydroxyl groups and, thereby, decreased the water interaction. As a result, there are less opportunities for water molecules to form bonds with the sorption sites, which also reduces the moisture absorption of the nanocomposite films. It was observed that the hydrophilic nature and morphological features of the nanocomposite films affected the moisture content at equilibrium. Higher BT concentrations were also associated with a declining trend in the films’ ability to swell, which was again explained by the interaction between the BT and carrageenan matrix as well as the filling in of cavities and crevices brought on by the rising clay content, which made the framework extra sturdy. Interestingly, while the moisture concentrations in the majority of the films decreased, the water contact angle values of the nanocomposite film surfaces decreased with increasing BT concentration, implying a stronger hydrophilic surface. This may be attributable to the ability of the nanoclay platelets to create a more compact structure, the polymeric matrix’s contact with the BT via H-bonding as well as some reconfiguration of the polymer network that the BT induced.

Zakuwan and Ahmad studied the impact of various reinforcements and filler compositions viz. cellulose nanocrystals and organically modified montmorillonite (OMMT) on the moisture absorption capabilities of carrageenan-based composites [4]. The capacity of OMMT to significantly enhance the polymer barrier properties has been demonstrated to be among the most important impacts on the attributes of the polymer matrix. Since OMMT sheets are inherently impervious, they generated a network of challenging pathways that slowed the diffusion of water molecules across the carrageenan matrix, creating a more hydrophobic nanocomposite.

According to a study by Alinavaz et al., who developed a variety of innovative nanomaterials based on the growth of calcium phosphate and hydroxyapatite (HA) within the polyvinyl alcohol/carrageenan (PVA/Car) hydrogels to evaluate their potency as drug delivery systems, the introduced bioceramics displayed an impact on the hydrophilicity of the carrageenan-based composite materials. PVA/Car with no integrated HA exhibited the highest degree of swelling; however, when HA was added, the swelling of hydrogel nanocomposites greatly reduced compared to the plain hydrogel, which is likely due to HA’s reduced ability to swell in comparison to the hydrogels. Additionally, there was less water absorption due to the physical interactions between the particles of hydroxyapatite and the matrix, specifically hydrogen bonds. These physical interactions may have also increased the number of crosslinking points, culminating in a higher crosslink density and, ultimately, a decreased porosity for water uptake in hydrogels [67].

#### 3.3.3. Metal Oxide Nanoparticles, Nanotubes and Carbon Dots

According to various reports, biopolymer-based films frequently incorporate nanomaterials as they can enhance the film’s characteristics and inculcate new functional features. It has typically been reported that the hydrophobic properties of nanofillers such as carbon dots, metal nanoparticles, and melanin have boosted the hydrophobicity of carrageenan film surfaces. The homogenous dissemination of nanoparticles inside the polymer matrix, which results in a sustained phase of a closely packed system between the hydrophobic nanoparticles and the carrageenan polymer matrix as well as the possibility of a dense structural system forming between the nanoparticles and the polymer matrix to restrict the penetrating course of water vapour have all been associated with a similar or reduced WVP of the nanocomposite films compared to neat carrageenan films [42,72,73].

According to the research findings, as shown in Table 9, the inclusion of titanium dioxide (TiO_2_) and titanium dioxide nanotubes (TNT) only mildly improved the WCA of carrageenan film (*p* > 0.05), but the addition of titanium dioxide nanotubes doped with copper oxide (TNT/CuO) substantially enhanced the WCA (*p* < 0.05). Additionally, TNT and TNT/CuO marginally decreased the WVP of the carrageenan film whereas TiO_2_ slightly increased it [42].

**Figure 6:**
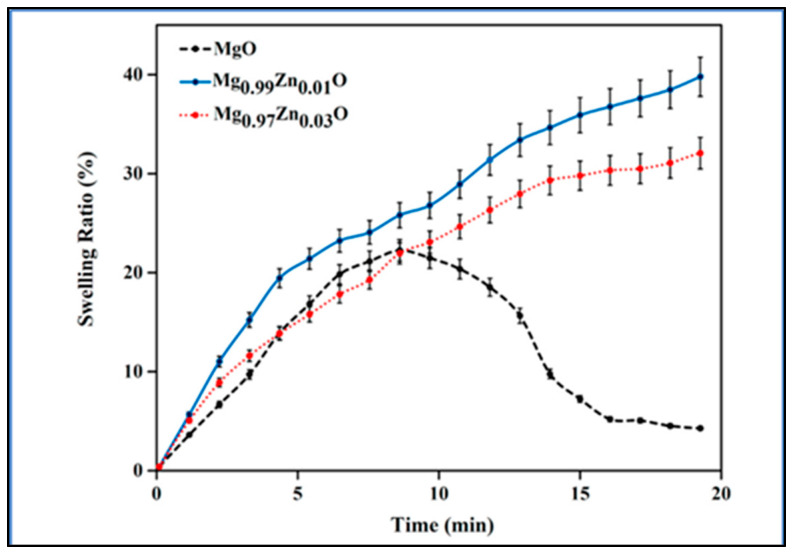
Swelling capacity of the kappa-carrageenan nanocomposite hydrogels containing MgO, Mg_0.99_Zn_0.01_O, and Mg_0.97_Zn_0.03_O (taken from [41] with permission from Elsevier).

Another study, as shown in Figure 6, demonstrated that increasing the zinc content from 0.01 to 0.03 mg led to a decline in the swelling ratio, which is attributable to the ability of ZnO nanoparticles to crosslink. The porosity would vary as the crosslinker content changed, which would have an impact on the swelling ratio [41].

A detailed tabulation (Table 10) of the effect of incorporated metal nanoparticles, nanotubes, and carbon dots on the water interactive properties of carrageenan-based composites is given below.

#### 3.3.4. Bioactive Agents

Developing biopolymer-based composites with the incorporation of bioactive agents has found extensive application in the field of food packaging as well as biomedicine. Carrageenan-based composite films combined with various types of such bioagents have been widely studied by several research groups across the world (Table 11).

**Figure 7:**
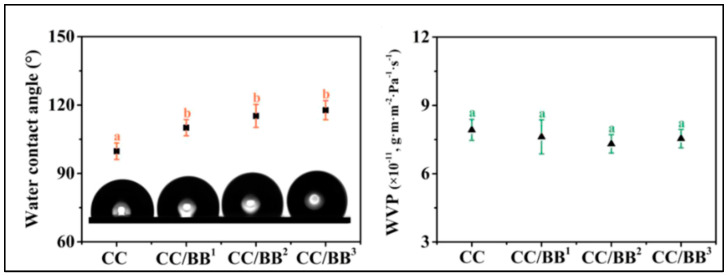
Water contact angle (WCA) values and the water vapour permeability (WVP) of sodium carboxymethylcellulose-carrageenan (CC) films alone and with self-assembled berberine-baicalin nanoparticles (CC/BB NPs) (taken from [80] with permission from the American Chemical Society).

According to one of the investigations by Li and co-workers, analogous composites with photodynamic antibacterial activity could be employed as potential food packaging. With the incorporation of self-assembled berberine-baicalin nanoparticles (CC/BB NPs), their analyses have revealed a considerable enhancement in the water contact angle of carrageenan/Na-carboxymethyl cellulose (CC) films, but not very substantial variations in the WVP measurements (Figure 7). According to the authors, the number of –OH groups that are available is decreased due to the hydrogen bonding force between the carrageenan and Na-CMC, which resulted in CC/BB films having an increased hydrophobicity than pristine carrageenan films. However, given that all of the films were composed of the same base material, it is possible that the identical WVPs could be associated with the pore structure. Furthermore, since the measured values were higher than those of the biopolymer-based films, CC/BB films could offer a potent water vapour barrier, minimising the moisture loss in the environment of packaged goods. It also stands to reason that the CC/BB film’s low WVP and hydrophobicity are synergistic, thereby reinforcing one another. The compact framework of the CC film and CC/BB films could further contribute to their improved water vapour barrier performance [80].

When Nouri and team looked at how the *Zataria multiflora extract* (ZME) affected the kappa-carrageenan-based films (Table 5 and Procedure 2 in Section 3.2.), they found that adding ZME reduced the WVP values of the composite films, with the 2% ZME loading percentage showing the greatest WVP reduction. Despite the fact that all of the components in these composite films were hydrophilic, the phenolic groups of ZME and the hydroxyl groups of glycerol participated in hydrogen bond formation with the functional groups of the carrageenan-nanoclay films, which reduced the number of hydroxyl groups available for interaction with the water molecules, which in turn resulted in reduced WVP values [55,82].

Souza et al. investigated the WCA on each of the various layers as they constructed and characterised multi-layered coatings composed of kappa-carrageenan (KC) and quercetin loaded lecithin/chitosan nanoparticles. They noticed that the quercetin nanoparticle-coated layer had WCA values around 50° whereas the KC layer showed values around 30°. They concluded that it was possible to modify the hydrophobicity or hydrophilicity of a multilayer system depending on the bioagent administered to the outermost surface [81].

#### 3.3.5. Synergistic Effects in Hybrid Bionanocomposites

Considering the features that result from the combinatorial effects that appear in the reinforcing zone, hybrid bionanocomposites have been utilised in a variety of fascinating applications in several fields. Superior mechanical capabilities and a reduction in moisture absorption characteristics have been clearly proven by studies on the hybridisation of nanofillers and natural fibres in various matrices (Table 12).

Amjadi and colleagues [56] measured the water contact angle of kappa-carrageenan (KC) composites with added zein nanofibres (Z), zinc oxide nanoparticles (ZnONPs), and rosemary essential oil (RE) to examine their synergistic effects on the surface hydrophobic nature. They found that the water contact angle values were significantly (*p* < 0.05) increased by the integration of ZnONPs and RE in the Z90/KC10 nanocomposite, with the greatest value attained at 77.7 ± 3.1°. The nanocomposite structure’s free hydrophilic groups were reduced by the hydrogen bonds that formed between zein, KC, and ZnONPs, increasing the material’s hydrophobicity. Additionally, the increased crystalline nature and compact size of the polymer chains could be accounted for by adding ZnONPs to the nanofibres, which enhanced the surface hydrophobicity values.

For use in the packaging of minced chicken meat, Praseptiangga and colleagues [2] developed and characterised semi-refined iota-carrageenan (SRiC)/SiO_2_–ZnO bionanocomposite sheets with added cassava starch (CS) with the following formulations. F0: SRiC film (control film), F1: SRiC film with SiO_2_–ZnO nanoparticles, F2: SRiC/CS (1.5 wt.%: 0.5 wt.%) with SiO_2_–ZnO nanoparticles, F3: SRiC/CS (1.0 wt.%: 1.0 wt.%) with SiO_2_–ZnO nanoparticles, F4: SRiC/CS (0.5 wt.%: 1.5 wt.%) with SiO_2_–ZnO nanoparticles. As evidenced by the AFM result, the critical surface tension of the F3 film was the highest and ascribed to the film’s surface roughness. Adhesiveness was more pronounced on the rough surface than cohesiveness, which made it easier for the liquid to saturate the film surface. The SiO_2_–ZnO nanoparticle performance was linked to the lowest WVP for F1 film, and the F1 and F2 films had a considerably (*p* < 0.05) higher water solubility than other films since SRiC is water-soluble due to its connection to the sulphate ester group. On the other hand, the amylopectin structure, which averts swelling and reduces water solubility, caused a significant (*p* < 0.05) drop in the water solubility of the films when the CS fraction was increased.

**Figure 8:**
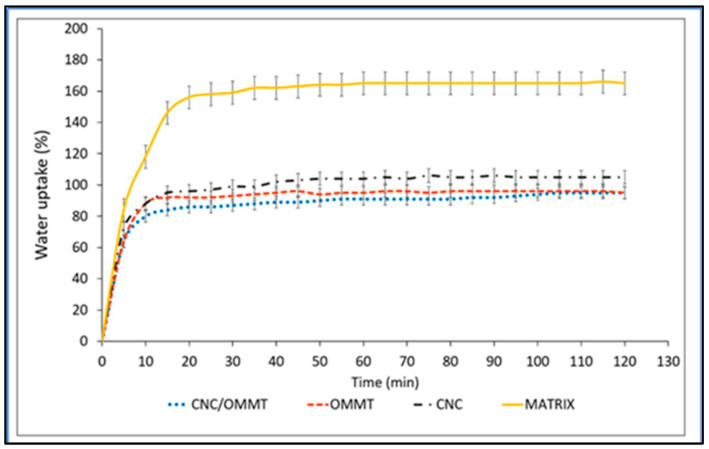
Water uptake of kappa-carrageenan matrix films: κ-carr matrix, κ-carr/CNC bionanocomposite, κ-carr/OMMT bionanocomposite, and hybrid κ-carr/CNC/OMMT bionanocomposite (Taken from [4], MDPI CC BY).

Vishnuvarthanan et al. designed carrageenan/silver nanoparticles/laponite nanocomposite coatings on oxygen plasma surface treated polypropylene for food packaging materials. According to their report, the WVTR of uncoated PP film was 9.62 g/m^2^/day and reduced to 7.65 g/m^2^/day after coating with carrageenan. The WVTR value decreased to 5.65 g/m^2^/day with the addition of 1 wt.% of laponite to carrageenan, which was further reduced to 5.58 g/m^2^/day following the inclusion of AgNPs. These results clearly establish the synergistic impacts of nanoclays and nanosilver, which caused a combinatorial effect in the decrease of WVTR values [1]. According to a study by Zakuwan and Ahmad, when the kappa-carrageenan films were reinforced using CNCs and OMMT, the water uptake was significantly reduced compared to the matrix without the filler by roughly 74% at 4% filler loading (Figure 8). This was predominantly due to the increased tortuosity produced by the dispersion of intercalated OMMT and the CNC structure, both of which absorbed free volume inside the matrix material and prevented water molecules from diffusing through the nanocomposites. It was observed that the morphology and type of polymer used in the production of the bionanocomposite materials had a significant impact on the diffusion coefficient. Thus, the combination of CNCs and OMMT showed great promise in addressing the drawbacks of kappa-carrageenan biopolymer films [4].

## 4. Conclusions

The moisture barrier property is one of the most predominant factors to be considered when measuring the effectiveness of a composite material for multifaceted applications. As the need for efficient and sustainable food quality and safety during storage and transportation grows as the food packaging business evolves, consumers, researchers, and the packaging industry are becoming more and more interested in biodegradable polymers made from renewable natural resources. Even though carrageenan is a suitable candidate for various applications in both food industry and biomedical fields, it can be understood that there exist a few limitations that hinder its efficiency depending on the end use applications. The safety concerns on the intake of carrageenan, not only in its degraded form, but also in its innate form, have posed substantial challenges in the widespread use of this versatile biopolymer. In order to combat the crisis of microbial contamination, food packaging with effective hydrophobicity and antibacterial capability is of great desire and demand. Therefore, hydrophobically modified carrageenan biocomposites could be utilised as an effective barrier packaging material to protect moisture sensitive products, not only fresh fruits and vegetables, but also processed and semi-processed food materials such as milk and milk products.

With this background understanding, the present review was put forward to gain a thorough understanding of the correlation between the formulation of carrageenan bionanocomposites and their hydrophobic nature. It was observed that different types of carrageenan (kappa or iota) as the matrix material played a role in their activity towards similar fillers, exhibiting slightly dissimilar trends in their hydrophobicity, whereas the refinement of carrageenan (refined and semi-refined) did not reveal any difference. The addition of plasticisers generally increased the hydrophilicity of the resultant composite materials, but was shown to be dependent on the preparation protocol. Polysaccharide-based nanoparticles as fillers resulted in an increase in hydrophobicity, which was also directly proportional to the filler concentration. In the case of nanoclays, bioceramics, and mineral NPs as fillers, higher hydrophobic values were observed irrespective of the composite forms (hydrogels, scaffolds and films), which also had a positive effect on antibacterial activity. Incorporation of metal oxide NPs, carbon nanotubes, and carbon dots improved the hydrophobicity and barrier ability, with significant improvement in mechanical properties and UV blocking capacity, while also imparting antimicrobial and the antioxidant properties to the bionanocomposite materials. It could also be observed that bioactive agents such as plant extracts, essential oils, etc. significantly increased the WCA values of the resultant composites while reducing their water vapour permeability. Such bionanocomposites also showed increased mechanical strength and a superior inhibition of bacterial growth and radical scavenging activity. Synergistic effects of two or more fillers were also reviewed, which revealed superior hydrophobicity, enhanced mechanical properties, increased cell viability, antimicrobial activity, and DPPH scavenging activity, both with individual fillers and in combination, while depending on the properties of the individual fillers.

Based on these recent studies, it can be understood that appropriate formulations can be developed to tackle the native hydrophilic nature of carrageenan and its composites, which could be a drawback in several applications, especially in the packaging industry. Hence, there exists room for further research in this field to improve the properties of carrageenan-based composites in order to expand its eclectic utility, either by functionalizing the polymer chains or by improvising suitable additives and compositions with the potential of hydrophobically modifying carrageenan composites without compromising their outstanding hallmark properties.

## Figures and Tables

**Table 1:** Search strategy and protocol.

Search Terms/Keywords	Carrageenan Bionanocomposites Hydrophobic modification Nanofillers Bioactive Agents
Databases	Google Scholar Science Direct Bielefeld Academic Search Engine (BASE) Semantic Scholar PubMed.gov
Publication types included	Research papers Work articles Conference proceedings
Publication types excluded	Review articles Book chapters
Language	English
Search period	2018–2023

**Table 2:** Effect of carrageenan type (kappa vs. iota) on the hydrophilicity of the composites.

No.	Type of Carrageenan	Nanofillers	Effect on Hydrophilicity of Composites	Effect on Other Properties of Composites	References
1.	Iota-carrageenan (Semi-refined)	Silicon dioxide and zinc oxide (SiO_2_ and ZnO)	Incorporation of nanoparticles (NPs) significantly decreased the water vapour permeability (WVP).	Increased ultraviolet (UV) screening and enhanced antimicrobial activity with the addition of NPs.	[2]
2.	Kappa-carrageenan (Semi-refined)	ZnO	Increased solubility (up to 92%) with 0.5% ZnO, followed by a reduction at higher ZnO% (up to 75%).	Tensile strength increased up to 32 MPa with 0.5% ZnO, but then reduced up to 23 MPa with increasing ZnO%. Elongation at break exhibited a reducing trend, with an insignificant difference.	[5]

**Table 3:** Effect of refined vs. semi-refined carrageenan with nanocellulose.

No.	Type of Carrageenan	Nanofillers	Effect on Hydrophilicity of Composites	Effect on Other Properties of Composites	References
1.	Refined and semi-refined kappa-carrageenan	Nanocellulose fibrils (NCF)	Lower moisture content (up to 23% for SRC and 25% for RC), higher water solubility (ranging between 38% and 60%), moisture uptake (between 47 and 67%), contact angle (between 38 and 100°) and WVP (between 8 and 16 g·mm·cm^−2^·h^−1^·Pa^−1^) for SRC + NCF than RC + NCF.	Overall properties of both semi-refined carrageenan (SRC) and refined carrageenan (RC) films were enhanced.	[51]
2.	Kappa-carrageenan	Cellulose nanocrystals (CNC) derived from Indian gooseberry pomace	Decreased WVP from 3.21 to 2.25 g·mm/m^2^·day·kPa with increasing CNC concentration from 1 to 7%.	No structural changes with added CNC. Increased tensile strength and crystallinity with increasing CNC concentration.	[39]
3.	Carrageenan	Agar, zinc sulphide nanoparticles (ZnS NP) and nanocellulose-based tea tree essential oil Pickering emulsion (TPE)	Addition of ZnSNP and TPE, alone or in combination slightly improved the water vapour barrier (WVP reduced from 0.66 to 0.52 × 10^−9^ g·m/m^2^·Pa·s) and water resistance of the films (WCA increased from 62 to 67°).	ZnSNPs improved mechanical strength, whereas PET slightly decreased the strength. However, the combined addition maintained the mechanical strength with slightly improved flexibility and thermal stability. Distinct antioxidant and antibacterial activity.	[52]
4.	Kappa-carrageenan	Cellulose nanocrystals	Decreased water uptake (from 15 to 10%) and water sorption (128 to 115%) properties, increased water contact angle (from 47 to 90°).	Not specified.	[53]
5.	Kappa-carrageenan	Cellulose nanocrystals	Better water barrier properties (water absorbency decreased from 886 to 562; film solubility decreased from 61 to 48; work of adhesion from 140 to 96), increased water contact angle (from 23 to 72°) and decreased water vapour permeation (from 8.9 to 4.7 × 10^−11^ g·m^−1^·s^−1^·Pa^−1^).	Superior mechanical, thermal, and UV barrier properties.	[36]
6.	Kappa-carrageenan	Cellulose nanocrystals (CNCs) and organically modified montmorillonite (OMMT)	Significant reduction in water uptake (from ~160 to ~85%), both with individual fillers and with their synergistic effect.	Enhanced mechanical properties.	[4]

**Table 4:** Effect of refined vs. semi-refined carrageenan with nanoclays.

No.	Type of Carrageenan	Nanofiller	Effect on Hydrophilicity of Composites	Effect on Other Properties of Composites	References
1.	Semi-refined kappa-carrageenan	Three types of nanoclays (Hydrophilic bentonite (HB), Cloisite 10A, Cloisite^®^ 30B)	Decreased WVP (12.6 to 12.2 × 10^−8^/g·mm·cm^−2^·h^−1^·Pa^−1^) with more hydrophobic nanoclay filler.	Semi-refined carrageenan more compatible with hydrophilic nanoclay, resulting in higher tensile and thermal properties. Hydrophobic nanoclay caused higher stiffness.	[54]
2.	Kappa-carrageenan	Bentonite nanoclay (BT)	Decrease of water contact angle (from 76 to 27°), water uptake (from 12 to 9%) and water sorption ability 128 to 121%) with increasing BT content.	Increased surface roughness. Did not induce exfoliation of bentonite layers into the matrix, but there was an intercalation of polymer chains between the clay sheets.	[43]
3.	Kappa-carrageenan	Nanoclay, *Zataria multiflora* plant extract (ZME) and glycerol (as plasticiser)	Increment in WVP with increasing ZME concentration according to Procedure 1 (1.21 to 2.21 × 10^−10^ g·m^−1^·s^−1^·Pa^−1^), and vice versa for Procedure 2 (2.83 to 1.54 × 10^−10^ g·m^−1^·s^−1^·Pa^−1^).	Incorporation of ZME resulted in strong UV screening effects. Increased film thickness and elongation at break (EB) values with increasing ZME concentration according to Procedure 1, and vice versa for Procedure 2. Tensile strength augmented with increasing ZME in both procedures.	[55]
4.	Kappa-carrageenan	Nanoclay (montmorillonite) and rosemary extract	Significant reduction in water vapour permeability (5.3 to 2.1 × 10^−10^ g/m·s·Pa).	Increased tensile strength and elongation at break. More than 99% inhibition against *B. cereus*, *E. coli*, *P. aeruginosa* and *S. aureus.*	[37]

**Table 5:** Effect of refined vs. semi-refined carrageenan with ZnO NPs.

No.	Type of Carrageenan	Nanofillers	Effect on Hydrophilicity of Composites	Effect on Other Properties of Composites	References
1.	Semi-refined kappa-carrageenan	ZnO nanoparticles	Increased solubility (up to 92%) with 0.5% ZnO, followed by reduction at higher ZnO% (up to 75%).	Tensile strength increased with 0.5% ZnO, but then reduced with increasing ZnO%. Elongation at break exhibited a reducing trend, with insignificant difference.	[5]
2.	Kappa-carrageenan	Sodium carboxymethyl cellulose (NaCMC) and Mg_1−x_Zn_x_O nanoparticles	Higher swelling ratio in the presence of Zn, but inversely proportional to its concentration (*Figure 6 in the cited article*).	Enhanced thermal stability, springiness, adhesion, and consistency. Reduced hardness.	[41]
3.	Kappa-carrageenan	ZnONPs/rosemary essential oil (RE)-incorporated zein nanofibres	Increased surface hydrophobicity (WCA increased from 33.8 to 77.7°) with the addition of ZnO and RE, both separately and together (additive effect).	Enhanced thermal and mechanical properties. Increased cell viability, antimicrobial activity and DPPH scavenging activity.	[56]
4.	Carrageenan	ZnO NP and m-ZnO NPs (capped/stabilized by melanin)	Increased water contact angle (63.8 to 73.7°) and increasing trend in WVP values (ranging between 1.58 and 1.22 × 10^−9^ g·m/m^2^·Pa·s).	Lesser transparency. Higher UV-blocking property, thermal stability, mechanical properties, and antibacterial activity.	[57]

**Table 6:** WVP values of the kappa-carrageenan (KC) composites via two different procedures (P1 and P2) (taken (abridged) from [55] with permission from Elsevier).

Film Type	WVP (g·m^−1^·s^−1^·Pa^−1^ × 10^−10^) via P1	WVP (g·m^−1^·s^−1^·Pa^−1^ × 10^−10^) via P2
Neat film (KC alone)	1.21	2.83
KC + 1% ZME	1.49	1.81
KC + 2% ZME	2.11	1.54
KC + 3% ZME	2.21	1.76

**Table 7:** Water interactive properties of the kappa-carrageenan/nanocellulose composites at different loading (taken from [36] with permission from Elsevier).

Sample Code	Water Absorbency	Film Solubility	WVP (×10^−11^ g·m^−1^·s^−1^·Pa^−1^)	Water Contact Angle (°)	Work of Adhesion
CNC0	885.94	60.94	8.93	23.30	139.69
CNC1	825.00	54.63	7.37	23.70	139.38
CNC3	821.43	53.97	6.25	46.45	122.97
CNC5	778.05	51.22	5.36	55.65	113.88
CNC7	761.54	49.23	4.69	57.75	111.60
CNC9	561.79	47.97	9.15	71.80	95.54

**Table 8:** Role of nanoclays, bioceramics, and mineral nanoparticles in the carrageenan nanocomposites.

No.	Form of Composites and End Use Applications	Components	Effect on Hydrophilicity of Composites	Effect on Other Properties of Composites	References
1.	Scaffolds for bone tissue engineering	Kappa-carrageenan and nano-hydroxyapatite and chitosan	Appropriate swelling ability.	Rough surface morphology, better interaction between the components, favourable crystallinity, and higher mechanical properties. Increased deposition of apatite layer and greater cell viability. Enhanced protein adsorption and favourable degradation rate.	[71]
2.	Hydrogels as potential drug delivery system	Kappa-carrageenan, polyvinyl alcohol and hydroxyapatite (bioceramic) nanoparticles	Lesser degree of swelling in presence of hydroxyapatite NPs (Decreased from 19.6 to 9.7 g/g).	Remarkable effect on antibacterial activity and in vitro release rate of ciprofloxacin.	[67]
3.	Films for active food packaging applications	Kappa-carrageenan and silver loaded aminosilane modified halloysite nanotubes	Enhanced WCA (55.3 to 69.7°) and reduced WVP (1.6 to 1.4 × 10^−9^ g·m/m^2^·Pa·s)	Increased UV-light barrier property and antibacterial activity.	[3]

**Table 9:** Mechanical and water interactive properties of the carrageenan composites with TiO_2_, TNT, and CuO (taken from [42] with permission from American Chemical Society).

Films	Thickness (μm)	TS (MPa)	EB (%)	EM (GPa)	WCA (deg)	WVP (×10^−9^ g·m/m^2^·Pa·s)
Carrageenan	53.3 ± 0.7 ^a^	45.5 ± 3.5 ^a^	3.3 ± 2.4 ^a^	1.9 ± 0.2 ^a^	59.6 ± 2.6 ^a^	
Car/TiO_2_	54.5 ± 1.3 ^a^	47.4 ± 1.9 ^a^	4.4 ± 1.1 ^a^	2.6 ± 0.3 ^a,b^	60.4 ± 1.6 ^a^	1.32 ± 0.25 ^a^
Car/TNT	62.8 ± 1.8 ^b^	55.8 ± 4.7 ^b^	4.2 ± 1.5 ^b^	3.1 ± 0.1 ^b^	62.3 ± 2.4 ^a^	1.19 ± 0.26 ^a^
Car/TNT−CuO	67.1 ± 4.1 ^b^	54.6 ± 2.1 ^b^	5.6 ± 1.3 ^a^	2.5 ± 0.4 ^a,b^	66.2 ± 3.3 ^b^	1.15 ± 0.01 ^a^

The values are presented as the mean ± standard deviation. Different letters within the same column indicate significant differences (*p* < 0.05) between treatment groups.

**Table 10:** Role of metal oxide nanoparticles, nanotubes, and carbon dots in the carrageenan nanocomposites.

No.	Form of Composites & End Use Applications	Components	Effect on Hydrophilicity of Composites	Effect on Other Properties of Composites	References
1.	Green halochromic smart and active packaging films	Kappa-carrageenan, gelatin, TiO_2_ nanoparticle and anthocyanin	Significant improvement in moisture resistance.	Enhanced UV–Visible light barrier property, increment in mechanical and bacteriostatic properties, inhibition of oxidative reactions. Decomposed within ∼30 days under simulated environmental conditions.	[74]
2.	Antimicrobial packaging films	Kappa-carrageenan and silver nanoparticles (AgNPs) prepared using pine needle extract-mediated synthesis	WVP and WCA significantly increased with added Ag NPs, but no significant increase as a function of increasing nanofiller concentration (except for 3% loading on WVP).	Film thickness, tensile strength. and elongation at break increased with added nanofiller, but independent of the loading concentration. Elastic modulus remained unchanged with 1% nanofiller loading, but increased with 2% and 3%. Significant improvement in UV blocking properties, antioxidant activity and potent antibacterial activity. Total colour difference (△E) of nanocomposite films increased significantly.	[45]
3.	Pseudo-pasteurization films for kumquat preservation	Kappa-carrageenan, ZnO-doped carbon nanoparticles (ZnO/C)	Improved hydrophobicity and barrier ability.	Outstanding antibacterial properties, enhanced preservation capacity, slight loss of colour and transparency of films, improved tensile strength, thermal stability to varying degrees, qualified safety of films (verified through haemolysis and cell cytotoxicity experiments).	[75]
4.	Films for active food packaging	Kappa-carrageenan, TiO_2_ nanotube (TNT), and CuO-doped TNT (TNT−CuO)	Increased surface hydrophobicity and water vapour barrier properties.	Imparted UV-blocking properties and increased mechanical strength. Significantly higher antibacterial activity for doped TNT than native TNT.	[42]
5.	Films for active food packaging applications	Carrageenan, gelatin, and Enoki mushroom-derived carbon dots (mCDs)	No significant changes in water vapour permeability and hydrophobicity.	Significant improvement in mechanical properties, strong antioxidant activity.	[76]
6.	Hydrogels to apply in biomedical research	Kappa carrageenan, NaCMC and Mg_1-x_Zn_x_O nanoparticles	Higher swelling ratio in the presence of Zn, but inversely proportional to its concentration.	Enhanced thermal stability, springiness, adhesion, and consistency, reduced hardness.	[41]
7.	Films for food packaging	Carrageenan and sulphur-coated iron oxide nanoparticles (Fe_3_O_4_@SNP)	Decreased WVP and increased water contact angle.	Effective UV blocking property and stronger antibacterial activity.	[40]
8.	Films for active packaging	Kappa-carrageenan and silver ion loaded 3-aminopropyl trimethoxysilane-modified Fe_3_O_4_ nanoparticles	Addition of Fe_3_O_4_ significantly reduced the WCA of films, while the addition of Fe_3_O_4_-Ag and Fe_3_O_4_@NH_2_-Ag reduced it to a lesser extent.	Stronger antimicrobial activity, improved thermal stability, and UV blocking properties.	[77]
9.	Films for active food packaging applications	Kappa-carrageenan and hybrid nanoparticles (HNPs) of Fe_3_O_4_@SiO_2_@PAMAM@AgNPs {PAMAM: Polyamidoamine}	Significant decrease in water contact angle, slight decrease in WVP.	Significant reduction in UV and visible light transmittance, clear antibacterial activity, increased thermal stability, slight increase in tensile strength (TS) and rigidity (EM), slight decrease in flexibility (EB).	[44]
10.	Films for applications in food and non-food industries as UV shielding packaging materials	Kappa-carrageenan, xanthan gum, gellan gum and TiO_2_	Increased contact angle, decreased moisture content and WVP upon increasing TiO_2_ content.	Tensile strength, tensile modulus, T_g_ and thermal stability greatly enhanced. Partial microbial activity against *S. aureus* and decreased UV light transmittance. The hydrophobic nature of TiO_2_ agglomerates reduces the integrity of the film structure.	[78]
11.	Nanocomposite coating on oxygen plasma surface modified polypropylene for food packaging	Kappa-carrageenan, silver nanoparticles and Laponite	Decreased water vapour transmission rate (WVTR).	Increased tensile and adhesion strength of the coated film, reduced OTR, strong antimicrobial activity.	[1]
12.	Films (Application not specified)	Kappa-carrageenan, gelatin and nano-SiO_2_	Decreased water vapour permeability, moisture content, and water solubility.	Film thickness not affected. Significant increase in tensile strength and Young’s modulus. Decreased oxygen permeability and increased turbidity.	[79]

**Table 11:** Role of bioactive agents in carrageenan-based bionanocomposites.

No.	Form of Composites and End Use Applications	Components	Effect on Hydrophilicity of Composites	Effect on Other Properties of Composites	References
1.	Films for safe and efficient antibacterial food packaging	Kappa-carrageenan, sodium carboxymethylcellulose and berberine−baicalin nanoparticles (BB NPs)	Significant increase in contact angle (from 99.7 to 117.8°) and slight decrease in WVP (∼7.92 × 10^−11^ g·m·m^−2^·Pa^−1^·s^−1^, with *p* > 0.05) (Figure 7).	Superior inhibition of bacterial growth, high ROS generation efficiency, enhanced transparency, UV-blocking performance, strong mechanical strength, thermal stability and oxygen barrier properties.	[80]
2.	Layer by layer coating for surface modifications in biomedical and food industry applications	Kappa-carrageenan and quercetin-loaded lecithin/chitosan nanoparticles	Oscillative behaviour in contact angles, with higher values on quercetin NP coated layers (~50°) and lower WCA values on carrageenan coated layers (~25°) (*Figure 2 in the cited article*).	Antioxidant capacity and DPPH radical scavenging activity. Devoid of cell toxicity.	[81]
3.	Films for active packing in food packing industry	Kappa-carrageenan, (fixed % of) nanoclay (montmorillonite) and (different % of) rosemary extract	Significant reduction in water vapour permeability (5.3 to 2.1 × 10^−10^ g/m·s·Pa).	Increased tensile strength and elongation at break. More than 99% inhibition against *B. cereus*, *E. coli*, *P. aeruginosa*, and *S. aureus*.	[37]

**Table 12:** Synergistic effects of various fillers in carrageenan-based biocomposites.

No.	Form of Composites and End Use Applications	Components	Effect on Hydrophilicity of Composites	Effect on Other Properties of Composites	References
1.	Films for minced chicken packaging	Semi-refined kappa-carrageenan, sorbitol, ZnO, SiO_2_ and cassava starch.	WVP decreased from 1.05 to 0.84 × 10^−6^ g^−1^·h^−1^·m^−1^·Pa^−1^, WCA increased from 98° to 133° and critical surface tension (CST) increased from 20 to 29 mN·m^−1^.	Thickness remained more or less the same, tensile strength slightly increased from 28.63 to 32.44 MPa and EAB showed an increasing trend.	[83]
2.	Films for active packaging applications	Carrageenan, agar, zinc sulphide nanoparticles, and nanocellulose-based tea tree essential oil Pickering emulsion (TPE).	Addition of ZnSNP and TPE, alone or in combination slightly improved the water vapour barrier (WVP reduced from 0.66 to 0.52 × 10^−9^ g·m/m^2^·Pa·s) and water resistance of the films (WCA increased from 62 to 67°).	ZnS NPs improved the mechanical strength, whereas PET slightly decreased the strength. However, the combined addition maintained the mechanical strength with slightly improved flexibility and thermal stability. Distinct antioxidant and antibacterial activity.	[52]
3.	Electrospun nanofibres to use as active layer in food packaging systems	Kappa carrageenan and ZnO NPs/rosemary essential oil (RE)-incorporated zein nanofibres.	Increased surface hydrophobicity (WCA increased from 33.8 to 77.7°) with the addition of ZnO and RE, both separately and together (additive effect).	Enhanced thermal and mechanical properties. Increased cell viability, antimicrobial activity and DPPH scavenging activity.	[56]
4.	Films for food packaging	Kappa carrageenan, cellulose nanocrystals (CNCs) and organically modified montmorillonite (OMMT).	Significant reduction in water uptake (from ~160 to ~ 85%), both with individual fillers and with their synergistic effect.	Enhanced mechanical properties.	[4]

## Data Availability

Data sharing not applicable.
